# CPT-11-Induced Delayed Diarrhea Develops via Reduced Aquaporin-3 Expression in the Colon

**DOI:** 10.3390/ijms19010170

**Published:** 2018-01-06

**Authors:** Risako Kon, Yuika Tsubota, Moe Minami, Saki Kato, Yukari Matsunaga, Hiroshi Kimura, Yuta Murakami, Tetsuya Fujikawa, Ryoya Sakurai, Rei Tomimoto, Yoshiaki Machida, Nobutomo Ikarashi, Kiyoshi Sugiyama

**Affiliations:** 1 Global Research Center for Innovative Life Science, Hoshi University, 2-4-41 Ebara, Shinagawa-ku, Tokyo 142-8501, Japan; r-kon@hoshi.ac.jp; 2 Department of Clinical Pharmacokinetics, Hoshi University, 2-4-41 Ebara, Shinagawa-ku, Tokyo 142-8501, Japan; 9reen.chem@gmail.com (Y.T.); k09m27r03a12@i.softbank.jp (M.M.); mil-ey.1123s@ezweb.ne.jp (S.K.); ruru.pink914@docomo.ne.jp (Y.M.); sadmj.jgj@icloud.com (H.K.); murakami-yuta@linical.co.jp (Y.M.); uitols@ybb.ne.jp (T.F.); wdpcbr@i.softbank.jp (R.S.); monari.come@icloud.com (R.T.); 3 Division of Applied Pharmaceutical Education and Research, Hoshi University, 2-4-41 Ebara, Shinagawa-ku, Tokyo 142-8501, Japan; y-machida@hoshi.ac.jp; 4 Department of Functional Molecular Kinetics, Hoshi University, 2-4-41 Ebara, Shinagawa-ku, Tokyo 142-8501, Japan

**Keywords:** aquaporin, CPT-11, colon, diarrhea, inflammation

## Abstract

While irinotecan (CPT-11) has a potent anti-cancer effect, it also causes serious diarrhea as an adverse reaction. In this study, we analyzed the pathogenic mechanism of CPT-11-induced delayed diarrhea by focusing on water channel aquaporin-3 (AQP3) in the colon. When rats received CPT-11, the expression level of AQP3 was reduced during severe diarrhea. It was found that the expression levels of inflammatory cytokines and the loss of crypt cells were increased in the colon when CPT-11 was administered. When celecoxib, an anti-inflammatory drug, was concomitantly administered, both the diarrhea and the reduced expression of AQP3 induced by CPT-11 were suppressed. The inflammation in the rat colon during diarrhea was caused via activated macrophage by CPT-11. These results showed that when CPT-11 is administered, the expression level of AQP3 in the colon is reduced, resulting in delayed diarrhea by preventing water transport from the intestinal tract. It was also suggested that the reduced expression of AQP3 might be due to the inflammation that occurs following the loss of colonic crypt cells and to the damage caused by the direct activation of macrophages by CPT-11. Therefore, it was considered that anti-inflammatory drugs that suppress the reduction of AQP3 expression could prevent CPT-11-induced delayed diarrhea.

## 1. Introduction

Irinotecan (CPT-11) is an anticancer agent that is widely used in the treatment of colon cancer, gastric cancer, pulmonary cancer, and cervical cancer. CPT-11 exerts its anticancer effects through the inhibition of DNA synthesis by inhibiting DNA topoisomerase I [1], but it also causes serious adverse drug reactions. The adverse reactions with high incidence include myelosuppression, nausea, vomiting, and diarrhea [2]. Among these reactions, diarrhea is a serious adverse drug reaction that occurs in approximately 80% of patients and is one of the dose-limiting factors of CPT-11 [3]. CPT-11-induced diarrhea is classified into early-onset diarrhea, occurring within a few hours of drug administration, and late-onset diarrhea, occurring more than 24 h after administration. Early-onset diarrhea is believed to be caused by the inhibition of acetylcholinesterase by CPT-11, leading to acetylcholine accumulation, as well as by the direct binding of CPT-11 to acetylcholine receptors, increasing peristaltic movement [4,5]. This early-onset diarrhea can be treated with anticholinergic agents such as atropine [6]. In contrast, late-onset diarrhea is very serious and is believed to be caused by the following mechanism. CPT-11 is metabolized mainly by carboxylesterases in the liver to form an active metabolite, SN-38, which is subsequently metabolized by uridine diphosphate-glucuronosyltransferase 1A1 (UGT1A1) into SN-38-glucuronide (SN-38-glu) [7,8]. After being excreted into the bile, SN-38-glu is deconjugated by enteric bacteria-derived β-glucuronidase in the intestinal tract and is re-converted into the active SN-38 [9,10]. This SN-38 is believed to induce severe, persistent diarrhea by damaging the intestinal mucosa and by accumulating in the body via the enterohepatic circulation [11]. Various methods to treat CPT-11-induced delayed diarrhea are being discussed based on this mechanism, including the following: (1) suppression of the production of SN-38 by killing enteric bacteria with antibiotics [12,13,14,15,16]; (2) inhibition of β-glucuronidase activity with Hangeshashinto, a traditional Kampo medicine [17,18]; and (3) adsorption of SN-38 in the intestinal tract using activated charcoal [19,20]. Other methods include the high-dose administration of loperamide, an antidiarrheal, as well as magnesium oxide, an antacid, to promote the excretion of SN-38 and CPT-11 by alkalizing the intestine [21,22]. Although these methods can alleviate diarrhea, treatment often becomes difficult in patients with severe diarrhea, and no reliable preventive methods have been established [23]. Severe, persistent diarrhea results in circulatory failure due to dehydration and electrolyte disturbance, which can then lead to death. Accordingly, it is crucial to suppress CPT-11-induced delayed diarrhea, not only because it improves the quality of life (QOL) of patients but also because it allows cancer treatment to be conducted smoothly.

In recent years, it has become clear that the water channel aquaporin (AQP) is expressed at significant levels in the colon, where the final fecal water content is controlled. In a previous study, we discovered that AQP3 is expressed at significant levels in colonic mucosal epithelial cells and that AQP3 plays a major role in the development of diarrhea and constipation [24,25,26,27]. These results led us to consider the possibility that AQP3 in the colon is involved in CPT-11-induced delayed diarrhea and that diarrhea may therefore be prevented from becoming severe by controlling AQP3 expression, thereby preventing dehydration. In this study, we investigated the mechanism of the development of CPT-11-induced delayed diarrhea by focusing on AQP3 to discover new preventive methods and/or treatments for CPT-11-induced delayed diarrhea.

## 2. Results

### 2.1. Effect of CPT-11 Dose and Administration Schedule on the Diarrhea Score and Mortality

To create a model for CPT-11-induced delayed diarrhea, the optimal dose and administration schedule of CPT-11 were investigated.

When CPT-11 was administered via the tail vein at various doses and schedules, early-onset diarrhea was observed within 3 h immediately after administration. Although no diarrhea was subsequently observed, it was found that soft stool and diarrhea started on the second day following the final administration of CPT-11, and most rats developed severe diarrhea by the third day. The diarrhea grade was calculated on the third day following the final administration. The results indicated that diarrhea did not develop in rats receiving CPT-11 at 60 or 80 mg/kg/day for four days, although soft stool was observed. In contrast, when CPT-11 was administered at 100 or 120 mg/kg/day for three or four days, most rats developed diarrhea. Although rats receiving CPT-11 at 120 mg/kg/day developed diarrhea at the same level as rats receiving 100 mg/kg/day, the associated weight loss was severe. In addition, rats receiving CPT-11 at 150 mg/kg/day exhibited high mortality (Table 1).

Based on the above results, it was found that the administration of CPT-11 at a dose of 100 mg/kg/day for four days causes a high incidence of diarrhea, while both weight loss and mortality remain low.

**Table 2:** Diarrhea scale.

Scale	Grade	Condition
0	Normal	Normal stool or absent
1	Slight	Slightly wet and soft stool
2	Moderate	Wet and unformed stool with moderate perianal staining of the coat
3	Severe	Watery stool with severe perianal staining of the coat

### 2.2. Evaluation of Diarrhea and Colitis in the CPT-11-Induced Delayed Diarrhea Rat Model

CPT-11 at a dose of 100 mg/kg was administered to rats via the tail vein for four days, and feces were collected on the third day following the last administration to investigate the level of diarrhea by analyzing the total weight of fecal pellets, total number of fecal pellets, and fecal water content. In addition, colitis was assessed by analyzing the condition of the colon tissue and inflammatory cytokines.

**Figure 1:**
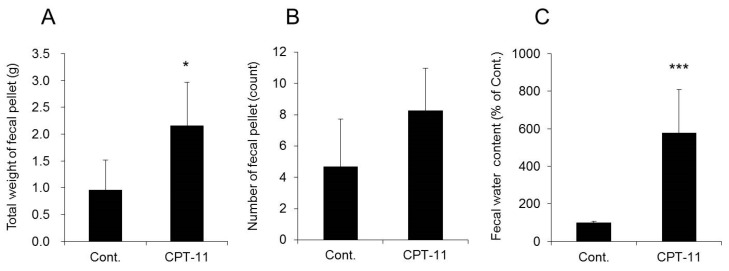
Assessment of diarrhea parameters after CPT-11 administration. CPT-11 (100 mg/kg/day) was administered in rats via the tail vein for 4 days. The total weight of fecal pellets (**A**), total number of fecal pellets (**B**), and fecal water content (**C**) were measured on the third day following the last administration. The fecal water content is shown with the mean value of the control group set at 100% (mean ± SD, *n* = 8, Student’s *t*-test: * *p* < 0.05, *** *p* < 0.001 vs. Cont.).

**Figure 2:**
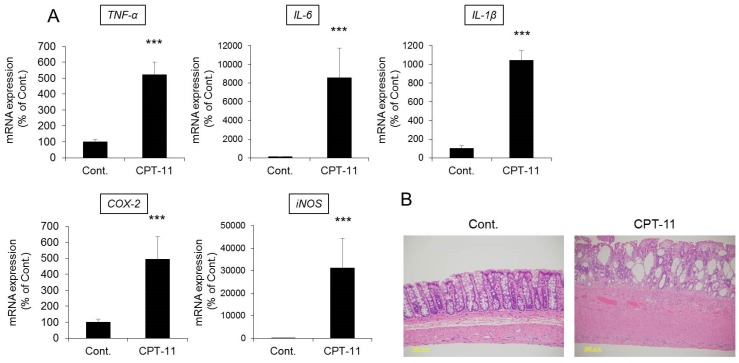
Changes in the inflammatory response and tissue morphology in the rat colon after CPT-11 administration. CPT-11 (100 mg/kg/day) was administered in rats via the tail vein for four days. The mRNA expressions of *TNF-α*, *IL-6*, *IL-1β*, *COX-2*, and *iNOS* in the colon were measured three days later using real-time polymerase chain reaction (PCR). After normalization with *β-actin*, data are presented with the mean value of the control group set at 100% (**A**) (mean ± SD, *n* = 8, Student’s *t*-test: *** *p* < 0.001 vs. Cont.). The colonic tissue morphology was observed by HE staining (**B**).

Both the total weight and fecal water content were significantly higher in rats in the CPT-11 administration group than in the rats in the control group, indicating the development of severe diarrhea (Figure 1). In addition, it was found that the mRNA expression levels of *cyclooxygenase-2* (*COX-2*), *inducible nitric oxide synthase* (*iNOS*), and inflammatory cytokines (e.g., *tumor necrosis factor-α* (*TNF-α*), *interleukin* (*IL*)*-6*, and *IL-1β*) increased significantly in the rat colon in the CPT-11 administration group (Figure 2A). The condition of the colon tissue at that time was investigated by hematoxylin and eosin (HE) staining, and both crypt cell damage and infiltration of inflammatory cells into the lamina propria were observed in the CPT-11 administration group. Fibroplasia and muscle layer thickness in the colon also increased in the CPT-11 administration group (Figure 2B). These changes were consistent with the characteristics of animal models of CPT-11-induced delayed diarrhea that have been previously reported [28,29].

Based on the above results, it was found that severe diarrhea develops in rats when CPT-11 is administered via the tail vein at a dose of 100 mg/kg/day for four days, at which point colitis is present.

### 2.3. Changes in AQP in the Colon in the CPT-11-Induced Delayed Diarrhea Rat Model

It has been reported that AQP1, AQP2, AQP3, AQP4, and AQP8 are found in the colon [30,31,32]. Therefore, we investigated the expression levels of these AQP using a rat model of CPT-11-induced delayed diarrhea.

**Figure 3:**
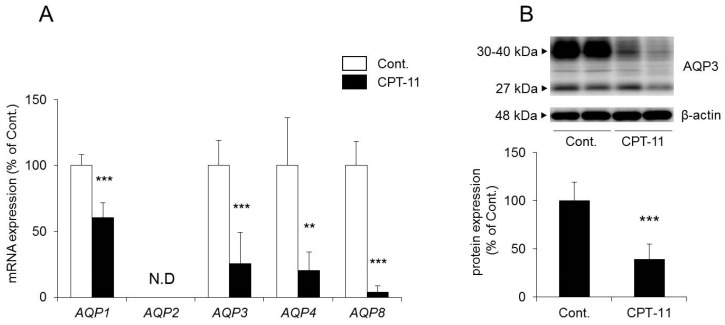
Change in AQP expression in the rat colon after CPT-11 administration. CPT-11 (100 mg/kg/day) was administered in rats via the tail vein for four days. The mRNA expressions of *AQP1*, *AQP2*, *AQP3*, *AQP4*, and *AQP8* in the colon were measured three days later using real-time PCR (**A**). The protein expression of AQP3 in the colon was analyzed by Western blotting (**B**). After normalization with β-actin, data are shown with the mean value of the control group set at 100% (mean ± SD, *n* = 8, Student’s *t*-test: ** *p* < 0.01, *** *p* < 0.001 vs. Cont.).

The mRNA expression levels of *AQP1*, *AQP3*, *AQP4*, and *AQP8* in the colon decreased significantly in rats in the CPT-11 administration group compared to the levels in the control group. No *AQP2* was detected (Figure 3A).

**Figure 4:**
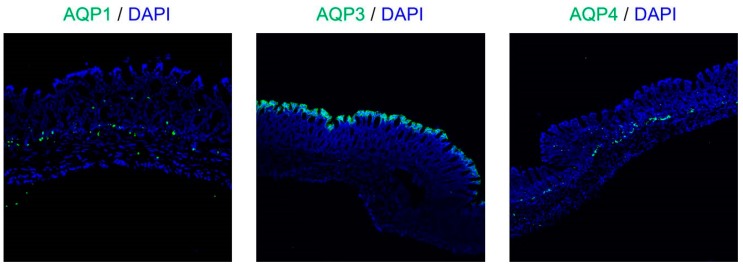
The distribution of AQP1, AQP3, and AQP4 in the normal rat colon. The AQP1, AQP3, and AQP4 (green) in rat colon were immunostained. The nuclei were counterstained with 4′,6-diamidino-2-phenylindole (DAPI; blue).

In a previous study, we found that AQP1 was expressed around blood vessels, AQP4 was expressed in muscular layer and AQP8 were low in the rat colon, using immunohistochemistry [24]. In this study, although we were not able to find AQP8, we found AQP1, AQP3, and AQP4 by immunohistochemistry (Figure 4). Among these, AQP3 was expressed especially in colonic mucosal epithelial cells. This distribution of AQP3 in rat colon is similar to this in human colon [32,33]. In addition, AQP3 plays a significant role in the development of diarrhea and constipation [24,25,26,27]. Therefore, we analyzed the protein expression level of AQP3 in colon membrane fractions by Western blotting. The results showed that AQP3 decreased significantly in rats in the CPT-11 administration group to approximately 40% of that in the control group (Figure 3B).

Based on the above results, it was found that the administration of CPT-11 markedly reduced *AQPs* in the colon. Specifically, AQP3 expression in mucosal epithelial cells was found to have decreased markedly, even at the protein level.

### 2.4. Effect of Celecoxib on the CPT-11-Induced Delayed Diarrhea Rat Model

Kase et al. reported that prostaglandin E_2_ (PGE_2_) production in the colon plays a major role in the development of CPT-11-induced delayed diarrhea and that Hangeshashinto, a traditional Kampo medicine, suppresses CPT-11-induced delayed diarrhea by reducing the production of PGE_2_ [34,35,36,37]. In addition, Trifan et al. reported that celecoxib, a selective COX-2 inhibitor involved in the production of PGE_2_, suppresses CPT-11-induced delayed diarrhea [38]. Therefore, we investigated whether CPT-11-induced delayed diarrhea and the reduced expression of AQP3 in the colon improved when celecoxib, an anti-inflammatory drug, and CPT-11 were administered in combination.

**Figure 5:**
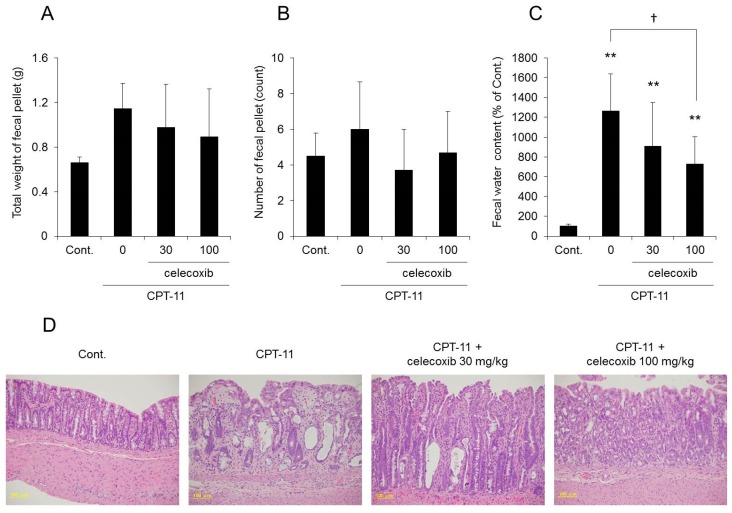
Effect of celecoxib on CPT-11-induced delayed diarrhea and colon tissue. CPT-11 (100 mg/kg/day) was administered to rats either alone or in combination with celecoxib. The total weight of the fecal pellets (**A**), total number of fecal pellets (**B**), and fecal water content (**C**) were measured on the third day following the last administration. The fecal water content is shown with the mean value of the control group set at 100% (mean ± SD, *n* = 8, Tukey’s test: ** *p* < 0.01 vs. Cont., † *p* < 0.05 vs. CPT-11). The colonic tissue was assessed by HE staining (**D**).

The fecal water content increased markedly in the group receiving CPT-11 alone compared with that in the control group, and all rats showed severe diarrhea, with a diarrhea score of three (Table 3). However, it was found that although the fecal water content was increased in the group receiving a combination of CPT-11 and celecoxib compared with that in the control group, it was lower than that in the group receiving CPT-11 alone (Figure 5C). In addition, when celecoxib was administered concomitantly, the diarrhea score decreased, and in particular, the incidence of severe diarrhea (grade 3) decreased (Table 3). This effect of celecoxib in improving diarrhea was found to be dose-dependent. When investigating the condition of the colon tissue, it was found that crypt cell damage, infiltration of inflammatory cells into the lamina propria, and edema caused by CPT-11 were reduced in a celecoxib dose-dependent manner (Figure 5D and Table 4).

**Figure 6:**
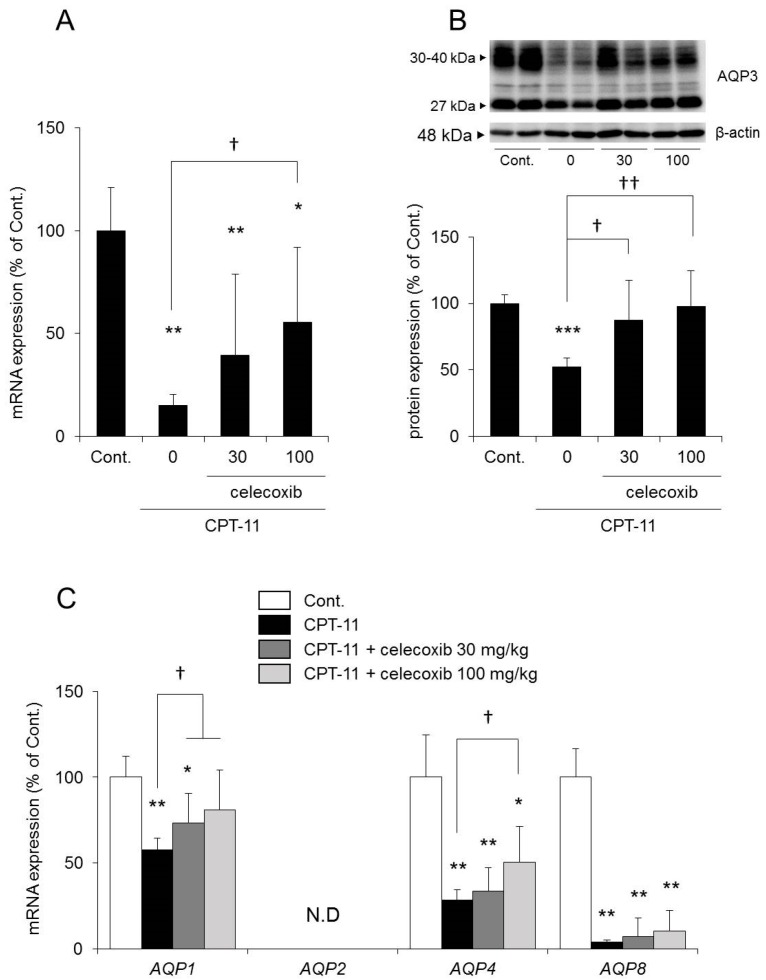
Effect of celecoxib on AQP expression in the rat colons of rats with CPT-11-induced delayed diarrhea. CPT-11 (100 mg/kg/day) was administered in rats either alone or in combination with celecoxib. The mRNA expression of *AQP3* in the colon was measured on the third day following the last administration by real-time PCR and was normalized with *β-actin* (**A**). The protein expression of AQP3 in the colon was analyzed by Western blotting and was normalized with β-actin (**B**). The mRNA expression of *AQP1*, *AQP2*, *AQP4*, and *AQP8* in the colon was analyzed by real-time PCR and was normalized with *β-actin* (**C**). Data are shown with the mean value of the control group set at 100% (mean ± SD, *n* = 8, Tukey’s test: * *p* < 0.05, ** *p* < 0.01, *** *p* < 0.001 vs. Cont., † *p* < 0.05, †† *p* < 0.01 vs. CPT-11).

The mRNA and protein expression levels of AQP3 in the colon were both significantly reduced in the group receiving CPT-11 alone compared with those in the control group. In contrast, the reduction in AQP3 expression was milder in the group receiving a combination of CPT-11 and celecoxib than in the group receiving CPT-11 alone, and the change was dependent on the dose of celecoxib (Figure 6A,B). In addition, the reduction in *AQP1* and *AQP4* mRNA expression by CPT-11 recovered after celecoxib administration. *AQP8* mRNA expression remained decreasing (Figure 6C). 

Based on the above results, it was found that the concomitant administration of celecoxib resulted in a decrease in the level of CPT-11-induced delayed diarrhea and a reduction in AQP3 protein expression in the colon.

### 2.5. Involvement of Colonic Macrophages in CPT-11-Induced Delayed Diarrhea

Based on the above results, it was found that the reduced expression of AQP3 in the colon plays a role in the development of CPT-11-induced diarrhea. In a previous study, we found that TNF-α and PGE_2_, which are secreted when colonic macrophages are activated, reduce the expression of AQP3 in mucosal epithelial cells in the colon [27]. Therefore, we investigated the mechanism of the reduction of AQP3 expression in the colon when CPT-11 is administered by focusing on the activation of macrophages.

**Figure 7:**
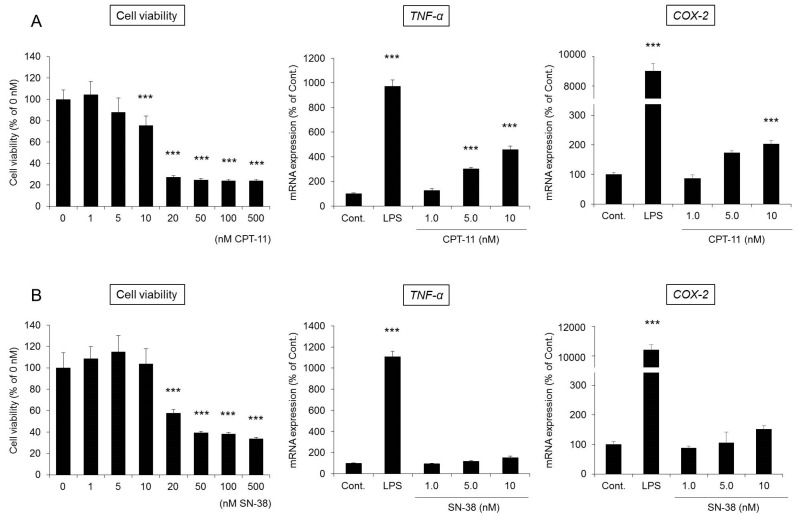
Effect of CPT-11 or SN-38 on RAW264 cell activation. CPT-11 (**A**), SN-38 (**B**), or lipopolysaccharide (LPS) as a positive control was added to RAW264 cells. After a 48-h incubation, cell viability was analyzed by the water-soluble tetrazolium salt (WST-1) assay. Data are shown with the mean value of the control group set at 100% (mean ± SD, *n* = 8, Dunnett’s test: *** *p* < 0.001 vs. Cont.). Cells were collected after a 48-h incubation, and the mRNA expression of *TNF-α* and *COX-2* was measured by real-time PCR. After normalization with *18S rRNA*, data are shown with the mean value for the control group set at 100% (mean ± SD, *n* = 4, Dunnett’s test: *** *p* < 0.01 vs. Cont.).

RAW264 cells are monocyte-derived macrophages and are frequently used to evaluate the functions of macrophages [39]. When RAW264 cells were incubated for 48 h after the addition of CPT-11 (0–500 μM) or SN-38 (0–500 nM), cytotoxicity began to be observed at 20 μM in the culture with CPT-11 and at 20 nM in the culture with SN-38, which is 1/1000 of the level in the CPT-11 culture (Figure 7). In addition, when the changes in the mRNA expression of *TNF-α* and *COX-2* were investigated at concentrations at which no toxicity was observed, significant increases in *TNF-α* and *COX-2* expression were observed only in the culture with CPT-11 (Figure 7A).

Based on the above observations, it was found that the cytotoxicity of SN-38 against macrophages was 1000 times greater than that of CPT-11. However, at concentrations at which no cytotoxicity was observed, CPT-11 exerted a more potent effect in activating macrophages. In addition, when CPT-11 or SN-38 was exposed for 12 h, no substantial degree of cytotoxicity or macrophage activation was observed.

## 3. Discussion

In this study, the mechanism of the development of CPT-11-induced delayed diarrhea was analyzed by focusing on AQPs, which are water channels expressed in the colon, to discover new preventive methods and treatments for the severe delayed diarrhea that occurs at the time of CPT-11 administration.

When CPT-11 was administered at 100 mg/kg/day for four days, severe diarrhea developed by the third day following the final dose, and the model showed the characteristics of CPT-11-induced delayed diarrhea (Figure 1) [28,29]. Next, AQP3 expression in the colonic mucosal epithelial cells in the model was analyzed. AQP3 in the CPT-11 administration group was markedly reduced at both the mRNA and protein levels (Figure 3). Based on this observation, it was hypothesized that when CPT-11 is administered, AQP3 is reduced, which then prevents water transport from the intestinal tract, resulting in retention of water in the colon and leading to the development of delayed diarrhea.

We investigated why AQP3 is reduced when CPT-11 is administered. It was previously believed that the mechanism of the onset of CPT-11-induced delayed diarrhea was damage to mucosal epithelial cells by the active metabolite SN-38 [11]. However, the colonic epithelial cells were present in our model rats (Figure 2B), and CPT-11 and SN-38 had little effect on AQP3 expression in an in vitro study (Figure S1). It was considered that the probability that mucosal damage is involved in the mechanism associated with the reduced AQP3 is low.

It has been reported that the levels of various inflammatory mediators, such as PGE_2_, are increased in the colon when CPT-11-induced delayed diarrhea develops [29,40,41]. In our model, the degree of CPT-11-induced delayed diarrhea was improved by the administration of celecoxib, an anti-inflammatory, and the reduced expression of AQP3 recovered to a level similar to that of the control (Figure 5 and Figure 6). These findings suggested that AQP3 plays a certain role in CPT-11-induced delayed diarrhea and that the increased production of PGE_2_ mediated by the increased COX-2 expression is involved in the reduced AQP3 expression. In addition, based on the results of the in vitro study, CPT-11 directly activates macrophages (Figure 7), and this action does not occur within a short period of time. Therefore, it was considered that the inflammation caused by the activation of macrophages and the accompanying reduced expression of AQP3 in the colon are characteristic of CPT-11-induced delayed diarrhea. Although the details of the mechanism of macrophage activation by CPT-11 are not clear, Li et al. reported that CPT-11 caused inflammation by activating NOD-like receptor protein-3 (NLRP3) and nuclear factor-kappa B (NF-κB) via c-Jun N-terminal kinase (JNK) in macrophages [42]. In addition, it is widely known that when cells are damaged, the production of TNF-α increases, causing inflammation. Therefore, it was considered that when CPT-11 was administered, the reduction of AQP3 expression was caused by the activation of macrophages by CPT-11 itself, as well as by inflammation that occurs following the loss of crypt cells.

The expression level of *AQP1*, *AQP4*, and *AQP8* in the colon was also reduced significantly in the CPT-11 administration group, as was that of AQP3 (Figure 3A). In the colon, AQP1 is expressed in vascular endothelial cells [43,44]. When AQP1 is reduced, it is believed that water in the colonic tissues cannot be transferred to blood vessels efficiently, and this may have caused the swelling of the colon observed in the CPT-11 administration group. Although AQP4 is found in the muscle layers of the colon, when an AQP4 knockout mouse and a transgenic mouse with an overexpression of muscle AQP4 were analyzed, neither showed a change in myofunction [45,46]. It has also been reported that AQP4 was reduced in regenerated muscle cells [47]. AQP4 may be reduced during muscle tissue regeneration caused by the proliferation of active myofibroblasts and by the increase in muscle layers and fibers triggered by chronic inflammation in the colon, which occur at the time of CPT-11 administration. AQP8 is expressed in the mucosal epithelial cells of the colon and Fischer et al. have suggested that AQP8 could be a marker protein for a normal large intestine [48]. Laforenza et al. also reported that AQP8 may play a role in water transport in the proximal colon [49]. However, the role of AQP8 in diarrhea or constipation has not been clarified completely. AQP3 is intensively expressed in the mucosal epithelial cells of the colon, and it was found that the regeneration of epithelial cells was advanced when inflammation was suppressed by celecoxib (Table 4). It has been reported that AQP3 not only functions as a water channel but is also involved in cell proliferation and cell migration [50,51]. Thiagarajah et al. reported that colitis became severe and that the regeneration of epithelial cells was delayed in AQP3 knockout mice [52]. Therefore, it was expected that an increase in AQP3 expression may not only attenuate CPT-11-induced diarrhea but may also play a major role in the regeneration of colonic tissues. In addition, recovery of the expression of other AQPs may also be useful in normalizing the swelling and muscle layers and in attenuating diarrhea.

In this study, the administration of celecoxib improved the reduction of AQP3 expression levels induced by CPT-11; however, it did not completely resolve the diarrhea (Table 3 and Figure 5C). The AQP3 localization in the CPT-11-treated group was similar to that in celecoxib-treated group (Figure S2). Therefore, we considered that the diarrhea was not resolved for the following reasons: (1) AQP8 might play a critical role in CPT-11-induced delayed diarrhea (Figure 6C) [49]; (2) CPT-11 activates cystic fibrosis transmembrane conductance regulator (CFTR), a chloride ion channel, which disrupts the balance of electrolyte transport in the colon [53]; (3) CPT-11 disturbs the composition of mucin, which is the mucus component that protects intestinal epithelial cells [54]; and (4) CPT-11 changes the intestinal microbiota [55]. In addition, it is known that at the time of CPT-11 administration, damage is observed not only in the colon but also in the lower section of the small intestine [28]. Therefore, to improve CPT-11-induced diarrhea, we considered that it is necessary not only to control the expression of AQP3 but also to improve these factors in an integrated manner.

In summary, AQP3 expression in the colonic mucosal epithelial cells is markedly reduced in CPT-11-induced delayed diarrhea. Celecoxib reduces CPT-11-induced delayed diarrhea. As celecoxib has been reported to enhance the anticancer effects of CPT-11 [38], celecoxib is considered useful in combination with CPT-11 as a symptomatic treatment. The results of this study also uncovered the potential of AQP3 in the colon as a new functional molecule in the mechanism of the development of CPT-11-induced delayed diarrhea. We consider the search for a comprehensive treatment to be crucial, including the control of AQP3 for the management of CPT-11-induced delayed diarrhea.

## 4. Materials and Methods

### 4.1. Materials

CPT-11 hydrochloride was purchased from Carbosynth Limited (Berkshire, UK). SN-38 was purchased from Tokyo Chemical Industry Co., Ltd. (Tokyo, Japan). Celecoxib was purchased from Combi-Blocks, Inc. (San Diego, CA, USA). d-sorbitol and TRI reagent were purchased from Sigma-Aldrich Corp. (St. Louis, MO, USA). Lactic acid and d-MEM medium were purchased from Wako Pure Chemicals (Osaka, Japan). WST-1 was purchased from Roche Diagnostics (Indianapolis, IN, USA). Rabbit anti-rat AQP3 antibody, rabbit anti-human AQP1 antibody, and rabbit anti-rat AQP4 antibody were purchased from Alomone Labs (Jerusalem, Israel). Rabbit anti-rat AQP8 antibody was purchased from Alpha Diagnostic Inc. (San Antonio, TX, USA). Alexa Fluor 488 donkey anti-rabbit IgG was purchased from Thermo Fisher Scientific Inc. (Waltham, MA, USA). Purified anti-β-actin antibody was purchased from BioLegend Inc. (San Diego, CA, USA). Donkey anti-rabbit IgG-HRP antibody and ECL prime Western blotting detection reagents were purchased from GE Healthcare (Chalfont St. Giles, UK). All primers for real-time PCR were purchased from Hokkaido System Science Co., Ltd. (Hokkaido, Japan). A high-capacity cDNA reverse transcription kit was purchased from Applied Biosystems (Foster City, CA, USA). SsoAdvanced SYBR green supermix was purchased from Bio-Rad Laboratories (Hercules, CA, USA).

### 4.2. Animals

Male Wistar rats (eight weeks old) were purchased from Japan SLC, Inc. (Shizuoka, Japan). The rats were housed at 24 ± 1 °C and 55 ± 1% humidity with 12 h of light (08:00–20:00). The study was conducted upon approval (approval No. 29–118, 29 June 2017) in accordance with the Hoshi University Guiding Principles for the Care and Use of Laboratory Animals.

### 4.3. Treatment

Rats were given lactic acid buffer (45 mg/mL d-sorbitol, 0.9 mg/mL lactic acid; pH 3.4) or CPT-11 (60, 80, 100, 120, or 150 mg/kg in lactic acid buffer) via the tail vein, and the level of diarrhea was evaluated on the third day following the final administration.

One day prior to the administration of CPT-11, rats were started on oral celecoxib (30 or 100 mg/kg/day in 0.5% methylcellulose) twice daily for eight days. CPT-11 (100 mg/kg in lactic acid buffer) was given via the tail vein for four days, and the colon was isolated under diethyl ether anesthesia on the third day following the final administration.

### 4.4. Assessment of Diarrhea

On the third day following the final administration of CPT-11, rat feces were collected to measure the total number of fecal pellets and total fecal weight. The fecal water content was calculated by freeze-drying the collected feces in a lyophilizer for 24 h, and the water content per gram of feces was calculated based on the moist weight and dry weight. The degree of diarrhea was assessed based on past reports (Table 2) [38].

### 4.5. HE Staining

The colons isolated from the rats were immersed in 10% neutral buffered formalin to fix the tissues. The tissues were embedded in paraffin and sectioned into 3 μm slices on glass slides. The slides were deparaffinized and stained with hematoxylin followed by eosin. The slides were dehydrated in alcohol, cleared in xylene, and covered for microscopic examination. The slides were read blindly by a pathologist and the colonic damage was assessed.

### 4.6. RAW264 Cell Culture

RAW264 cells (Riken Cell Bank, Tsukuba, Japan) were cultured using d-MEM medium (100 U/mL penicillin G potassium, 100 μg/mL streptomycin, and 10% fetal bovine serum).

RAW264 cells were plated in a 96 well-plate at a cell density of 1 × 10^4^ cells/well and incubated for 24 h. After CPT-11 (0–500 μM) or SN-38 (0–500 nM) was added, cells were incubated for 48 h, and cell viability was measured using the WST-1 assay. RAW264 cells were also plated in a 12 well-plate at a cell density of 2 × 10^5^ cells/well and incubated for 24 h. The expression level of each gene was analyzed using cells that had been incubated for 48 h after the addition of LPS (10 ng/mL), CPT-11 (0–10 μM) or SN-38 (0–10 nM).

### 4.7. WST-1 Assay

RAW264 cells were plated in a 96 well-plate and incubated for 48 h after the addition of drugs. After washing each well, WST-1 was added at a ratio of 10/100 μL medium, and cells were incubated for 2 h at 37 °C in a CO_2_ incubator. The absorbance at 450 nm was measured using a microplate reader.

### 4.8. Total RNA Preparations and Real-Time RT-PCR

TRI reagent was added to colon tissue or RAW264 cells, and total RNA was extracted. A high-capacity cDNA reverse transcription kit was used to synthesize cDNA from 1 μg of RNA.

Real-time PCR was performed using the primers listed in Table 5 and the following mixture: 2 μL of cDNA solution (2.5 ng/μL), 0.6 μL of forward primer (5 pmol/μL) and reverse primer (5 pmol/μL), 5 μL of SsoAdvanced SYBR Green Supermix, and 2.8 μL of RNase-free water. The reaction conditions included denaturation at 95 °C for 15 s, annealing at 56 °C for 30 s, and elongation at 72 °C for 30 s. The fluorescence intensity of the amplification process was monitored using the CFX Connect™ Real-Time PCR Detection System (Bio-Rad Laboratories). *β-Actin* and *18S rRNA* expression levels in reagent-treated group and control group did not differ.

### 4.9. Extraction of the Plasma Membrane Fraction from the Rat Colons

The mucosa was scraped from each rat colon sample, suspended in dissecting buffer (0.3 M sucrose, 25 mM imidazole, 1 mM ethylenediaminetetraacetic acid, 8.5 μM leupeptin, and 1 μM phenylmethylsulfonyl fluoride; pH 7.2) and homogenized on ice. The homogenate was centrifuged (800× *g* at 4 °C for 15 min), and the resulting supernatant was further centrifuged (17,000× *g* at 4 °C for 30 min). The supernatant was removed, and dissecting buffer was added to the precipitate, which was then dispersed using an ultrasonic homogenizer. This solution included the plasma membrane fraction with abundant cell membranes [26,56].

### 4.10. Western Blotting

Protein concentrations were measured by the bicinchoninic acid methods using bovine serum albumin as standard. Each sample was diluted with loading buffer (84 mM Tris, 20% glycerol, 0.004% bromophenol blue, 4.6% sodium dodecyl sulfate, and 10% 2-mercaptoethanol; pH 6.8), and samples were loaded in each lane. After polyacrylamide gel electrophoresis, the proteins were transferred to a polyvinylidene difluoride membrane. After blocking with skim milk, the resulting membrane was incubated with rabbit anti-rat AQP3 or anti-β-actin antibody for 1 h, followed by washing and incubation with donkey anti-rabbit IgG-HRP antibody for 1 h. The membrane was washed and then reacted with the ECL prime Western blotting detection reagents, and the bands detected by the LAS-3000 mini-imaging system (FUJIFILM, Tokyo, Japan) were analyzed.

### 4.11. Immunohistochenistry

The colon was post-fixed in 4% paraformaldehyde. The tissues were embedded, and the frozen blocks were sectioned into 10 μm slices on glass slides. The sections were reacted with a rabbit anti-human AQP1 antibody, rabbit anti-rat AQP3 antibody, rabbit anti-rat AQP4 antibody, and rabbit anti-rat AQP8 antibody. The sections were treated with an Alexa Fluor 488 donkey anti-rabbit IgG antibody. The slides were covered and observed under a fluorescent microscope.

### 4.12. Statistical Analysis

Numerical data are expressed as the mean ± standard deviation (SD). The significance of the differences was examined using Tukey’s test and Student’s *t*-test.

## Figures and Tables

**Table 1:** Effect of CPT-11 dose and administration schedule on the diarrhea grade and mortality.

CPT-11 Dose (mg/kg/day)	Schedule	Total CPT-11 (mg/kg rat)	Average Diarrhea Grade	Body Weight Loss (% of Control)	Mortality (%)
60	4 day	240	0.2	5.1	0
80	4 day	320	1.25	11.8	0
100	3 day	300	2	16.1	0
100	4 day	400	2.5	23.3	0
120	3 day	360	2.5	26.7	0
120	4 day	480	2.7	25.2	0
150	1 day	150	0	0	50
150	2 day	300	0	0	100

CPT-11 (60, 80, 100, 120, or 150 mg/kg in lactic acid buffer) was administered in rats via the tail vein. The grade of diarrhea was evaluated on the third day following the final administration using the diarrhea scale (Table 2). The rates of weight loss were measured with the control group value set at 100% (*n* = 4).

**Table 3:** Effect of celecoxib on CPT-11-induced diarrhea.

Group	Incidence of Diarrhea Scale		Average Diarrhea Grade	Body Weight Loss (% of Control)
	Grade 2 (%)	Grade 3 (%)		
Control	0	0	0	0
CPT-11 alone	0	100	3	28.5
+ celecoxib 30 mg/kg	42.9	57.1	2.6	26.7
+ celecoxib 100 mg/kg	83.3	16.7	2.2	28.0

CPT-11 (100 mg/kg/day) was administered in rats either alone or in combination with celecoxib. The level of diarrhea was evaluated on the third day following the final administration of CPT-11 using the diarrhea scale (Table 2), and the rate of weight loss was also investigated at that time (*n* = 8).

**Table 4:** Effect of celecoxib on colonic damage by CPT-11.

Colon Condition	Control	CPT-11 Alone	CPT-11 + Celecoxib	
			30 mg/kg	100 mg/kg
Disruption of epithelial cells	−	−	−	−
Disruption of crypt cells	−	++	+	+
Inflammatory cell infiltration	−	++	++	+
Swelling	−	+	−	−
Regeneration of epithelial cells	−	++	+++	+++

CPT-11 (100 mg/kg/day) was administered in rats alone or in combination with celecoxib. The tissue condition of the colon was assessed by HE staining on the third day following the last administration.

**Table 5:** Primer sequences for real-time PCR.

Gene	Forward (5′-3′)	Reverse (5′-3′)
r*TNF-α*	GAAACACACGAGACGCTGAAGT	CACTGGATCCCGGAATGTCGAT
r*IL-1β*	TCAGGCTTCCTTGTGCAAGTGT	ACAGGTCATTCTCCTCACTGTC
r*IL-6*	TAGTCCTTCCTACCCCAACTTC	GCCGAGTAGACCTCATAGTGAC
r*COX-2*	GCTGATGACTGCCCAACTC	GATCCGGGATGAACTCTCTC
r*iNOS*	CAAGCACATTTGGCAATGGA	GCCAAATACCGCATACCTGA
r*AQP1*	CTGGTGCTGTGCGTTCTG	GTCCAAGAGCCACAGACAAG
r*AQP2*	GGTTCCCAGTGCAGAGTAG	GAGGGTAGCTCAAGGCTTC
r*AQP3*	CCCCTTGTGATGCCTCTC	CCCTAGCTGGCAGAGTTC
r*AQP4*	AGGAGGACCCAGGCAATG	GGCAAGGTCTCATGCCATC
r*AQP8*	TTGGGCTCCGCTCTCTTC	CAAGGCCAGCCCATGAG
r*β-actin*	GCCACTGCCGCATCCTCTTG	CGGAACCGCTCATTGCCGAT
m*TNF-α*	ATGGACACCAAACATTTCCTGC	CCAGTGGAGAGCCGATTCC
m*COX-2*	CAGGGCCCTTCCTCCCGTAG	GCCTTGGGGGTCAGGGATGA
m*18S rRNA*	GTCTGTGATGCCCTTAGATG	AGCTTATGACCCGCACTTAC

## Supplementary Materials

Supplementary materials can be found at www.mdpi.com/1422-0067/19/1/170/s1.

## Abbreviations

AQP	Aquaporin
CFTR	Cystic fibrosis transmembrane conductance regulator
COX	Cyclooxygenase
CPT-11	Camptothecin-11, irinotecan
DAPI	4′,6-diamidino-2-phenylindole
HE	Hematoxylin and eosin
IL	Interleukin
iNOS	Inducible nitric oxide synthase
JNK	c-Jun N-terminal kinase
LPS	Lipopolysaccharide
NF-κB	Nuclear factor-kappa B
NLRP3	NOD-like receptor protein-3
PCR	polymerase chain reaction
PGE	Prostaglandin E
QOL	Quality of life
SN-38	7-ethyl-10-hydroxy-camptothecin
SN-38-glu	7-ethyl-10-hydroxy-camptothecin glucronide
TNF	Tumor necrosis factor
UGT	Uridine diphosphate-glucuronosyltransferase
WST-1	Water-soluble tetrazolium salt
