# Supplementary material for: CPT-11-Induced Delayed Diarrhea Develops via Reduced Aquaporin-3 Expression in the Colon
Source: Int J Mol Sci. 2018 Jan 6;19(1):170. doi: 10.3390/ijms19010170 (PMC5796119; doi:10.3390/ijms19010170)
Supplement: Supplementary file 1 [file ijms-19-00170-s001.pdf]

Supplementary Materials: CPT-11-Induced Delayed Diarrhea Develops via Reduced Aquaporin-3 Expression in the Colon

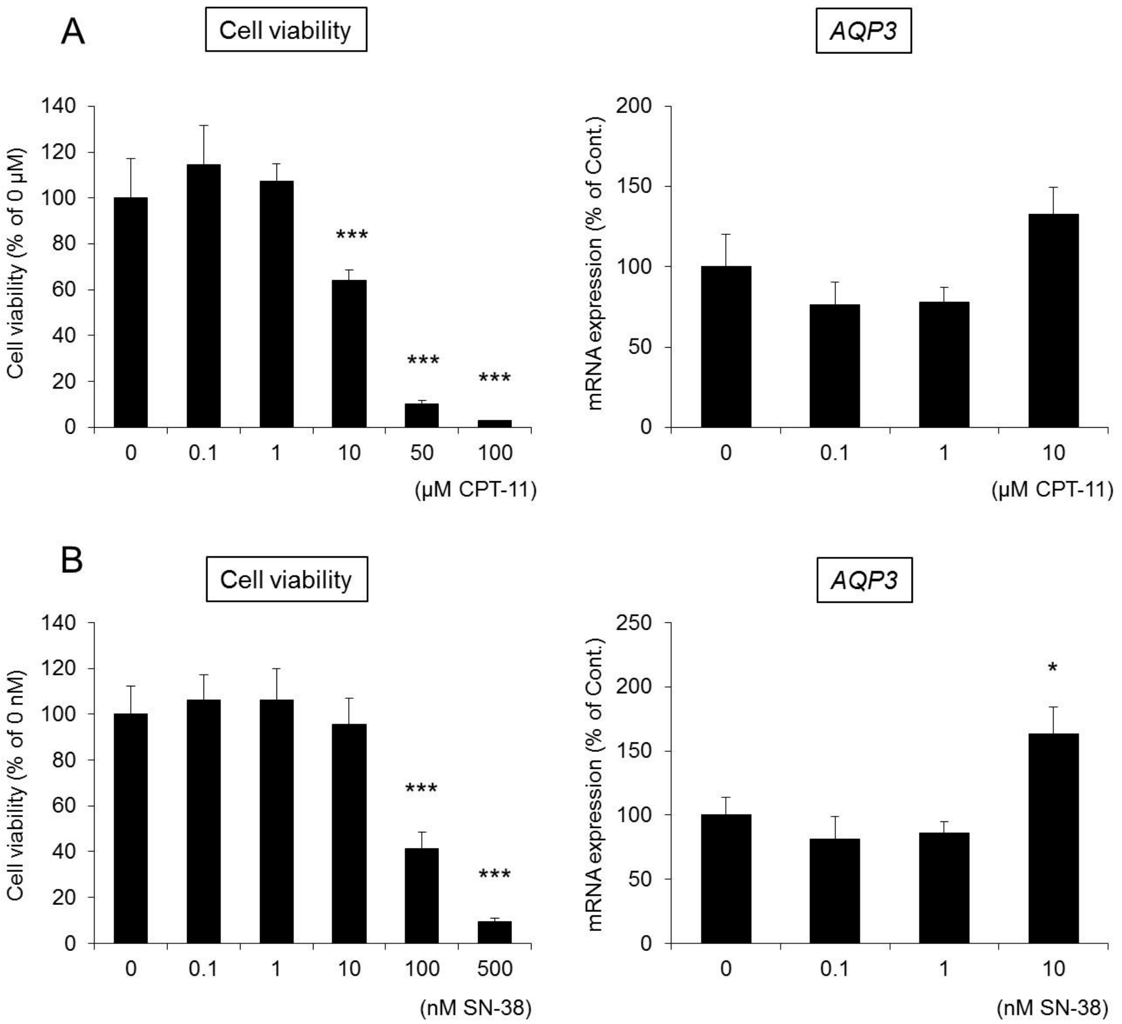

Figure S1. Effect of CPT-11 or SN-38 on human colon cancer HT-29 cells. HT-29 cells were treated with CPT-11 (**A**) or SN-38 (**B**). After a 48-hour incubation, cell viability was analyzed by WST-1 assay. Data are shown with the mean value of the control set at 100% (mean ± SD, n = 8, Dunnett's test: *** $p < 0.001$ vs. Cont.). Cells were collected after a 48-hour incubation, and the *AQP3* mRNA expression was measured by real-time PCR. After normalization with *β-actin*, data are shown with the mean value for the control set at 100% (mean ± SD, n = 4, Dunnett's test: * $p < 0.01$ vs. Cont.).

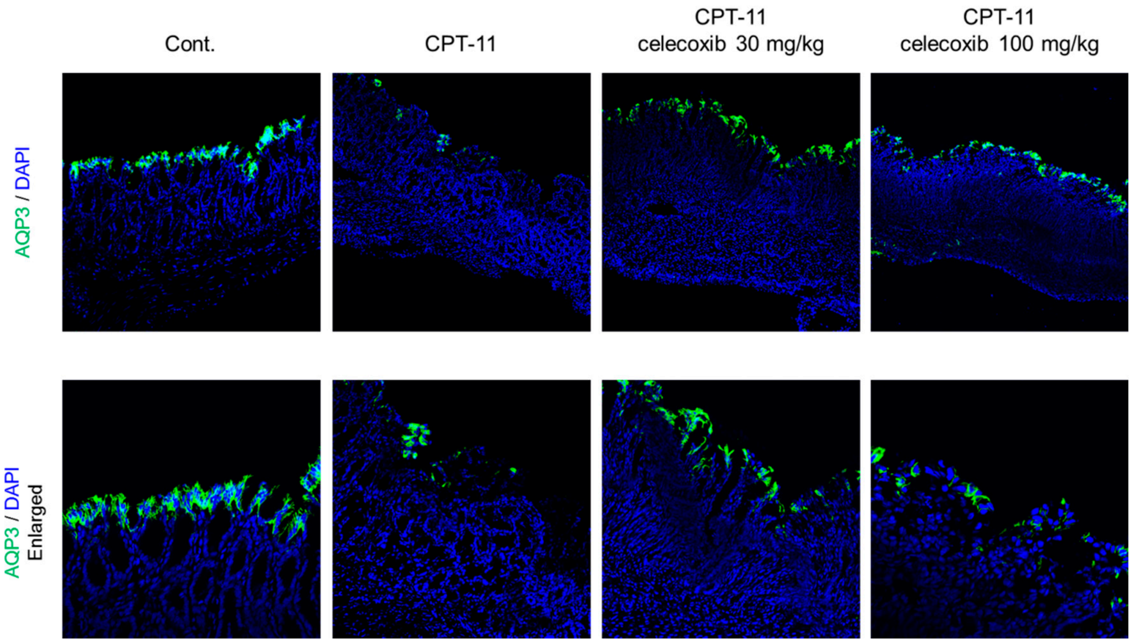

Figure S2. The distribution of AQP3 in the rat colon after treatment. CPT-11 (100 mg/kg/day) was administered to rats either alone or in combination with celecoxib. The AQP3 (green) in rat colon were immunostained. The nuclei were counterstained with DAPI (blue).
